# Meta-analysis of exercise intervention on health behaviors in middle-aged and older adults

**DOI:** 10.3389/fpsyg.2023.1308602

**Published:** 2024-02-28

**Authors:** Min Liu, Dong-hui Mei, Ya-lu Zhang, Ning Kang, Dong-min Wang, Gong Chen

**Affiliations:** ^1^Institute of Population Research, Peking University, Beijing, China; ^2^School of Social Welfare, Stony Brook University, Stony Brook, NY, United States; ^3^Department of Physical Education, Peking University, Beijing, China

**Keywords:** exercise intervention, middle-aged and older adults, health behavior, systematic evaluation, meta-analysis

## Abstract

**Objective:**

To systematically review and analyze the effects of exercise interventions on health behavior among middle-aged and older adults.

**Methods:**

A Meta-analysis was performed using NoteExpress software to screen randomized controlled trials (RCTs) published between January 1, 2000, and March 31, 2023, which were identified through databases including CNKI, Web of Science, Pubmed, and 6 more sources, based on predefined literature inclusion criteria. Following the quality assessment, we conducted both the overall and subgroup Meta-analyses of the exercise intervention moderator variables using Review Manager 5.4.1 software, encompassing data among the 18 RCTs. The effect size was measured as the standardized mean difference within its corresponding 95% confidence interval. Heterogeneity was assessed using the *I*^2^ metrics.

**Results:**

(1) The overall results indicate a significant impact of exercise intervention on health behaviors, characterized by a large effect size [SMD = 1.02 > 0.8, 95% CI (0.73, 1.32), *P* < 0.001]. (2) The highest degree of heterogeneity in the relationship between exercise interventions and health behaviors was associated with the duration of one exercise session (*I*^2^= 71.2%), which was the most influential moderator variable. (3) The aerobic and resistance intervention in group exercise lasting 30–60 min per time a day, 6–7 times per week over a period of 8–12 weeks demonstrated the most substantial effect size.

**Conclusion:**

(1) The exercise intervention significantly promotes the health behavior in middle-aged and older adults, emphasizing the importance of carefully considering the duration of individual exercise sessions when designing and implementing exercise intervention. (2) Considering the accessibility for middle-aged and older adults, the optimal exercise intervention should include the means of group practice, the types of aerobic and resistance exercise, with a duration of 30–60 min per time a day, beginning with a lower weekly frequency and gradually increase to 6–7 times per week, and lasting for 8 to 12 weeks.

**Systematic Review Registration:**

https://www.crd.york.ac.uk/prospero/, identifier CRD42024506750.

## 1 Introduction

Globally, the process of population aging is steadily advancing, with a growing proportion of middle-aged and older adults. According to the 2022 data from the World Population Prospects, the proportion of adults aged 45 and above globally accounts for 30.48%, marking a 2.88% increase compared to 2013 (United Nations, 2022). Middle-aged and older stages of life represent crucial phases in the human life cycle (Sharifi et al., 2014). During this period, metabolic rates, physical fitness, and musculoskeletal joints functions naturally decline (Kang et al., 2022). The health status of middle-aged and older adults plays a pivotal role in addressing the challenges of population aging. Promoting health-conscious behaviors among individuals in these age groups holds particular significance, as it substantially impacts the enhancement of their overall well-being (Gao, 2015). Under the background of global population aging, the 30^th^ World Health Assembly explicitly pointed out *Health for All by the* Year 2000 *(HFA/2000)*, which is a pivotal benchmark in global health strategy. The strategy stated that a new way of life and new opportunities is required to realize a higher standard of health. Therefore, it is a compelling need to explore significant intervention methods and strategies to improve the health behaviors of middle-aged and older adults.

The concept of “health behavior” was initially introduced by Kasl and Cobb in 1966 (Kasl and Cobb, 1966). In the field of behavioral science, “behavior” refers to the thought-oriented subjective performance undertaken by individuals in response to stimuli (Cao, 1987). “Healthy behavior” encompasses a series of preventive and protective measures designed to prevent or detect diseases early, with the aim of maintaining and promoting one’s level of health (Tatlor, 1986; Steptoe et al., 1997). Health behaviors pertain various aspects such as exercise routine habits, emotional well-being, dietary choices, and sleeping patterns (Glanz and Oldenburg, 1997). While these aspects can be individually assessed, the results are presented as independent scores rather than a cumulative health behavior score. The total health behavior score serves as a comprehensive index for assessing the overall health behavior of middle-aged and older adults. Therefore, in order to gain a comprehensive understand of health behavior, universal scales are frequently used (Song and Lee, 2001; Wang et al., 2010; Dong et al., 2022; Yu and Xu, 2022) for an overall evaluation. A higher total score indicates a greater engagement in health behavior and a higher overall level of health behaviors.

Intervention has the potential to change willingness and behavior. As mentioned above, exercise routine habit is one of the dimensions of healthy behavior. However, it is important to distinguish between exercise habit and exercise intervention. The former is a subjective manifestation of voluntary willingness, emphasizing proactive engagement rather than passive acceptance. Nevertheless, the latter entails highly participatory means to improve individuals’ involvement, even when they may not have initially volunteered. Unlike medical treatments, it can bring about improvements without the need for other substances to enter the body (Dulfer et al., 2013; Fife-Schaw et al., 2014; Martín-García et al., 2019; Smith et al., 2020; Browne et al., 2021; Stathi et al., 2022).

Meta-analysis, a solid methodology to assess the results systematically and quantitatively obtained in evidence-based studies in medicine and social science, yields high-quality research conclusions obtained by integrating the systematic evaluation of data results with shared purpose and similar properties. But in fact, few studies have explored the impact of exercise interventions on health behaviors among middle-aged and older adults at the level of systematic evaluation. Among the existing Meta-analysis studies investigating the effect of exercise interventions on health behaviors, Bentley, Mitchell, and Backhouse (Bentley et al., 2020) explored the effect of exercise interventions on dietary structures. Gür and Can Gür (2020) performed a Meta-analysis on exercise’s effects to alcohol consumption in adults. Shi et al. (2022) explored the effects of exercise interventions on smoking cessation. Peng et al. (2022) conducted a systematic evaluation of exercise interventions on enhancing the physical activity level in the general population. Gong et al. (2021) and Du et al. (2015) examined the effects of physical exercise on improving sleep quality.

Each of the aforementioned studies have primarily addressed the significance of exercise intervention in promoting one specific aspect of health behaviors, rather than comprehensively assessing overall health behavior levels. What’s more, the optimal intervention methods, modes, specific exercise types, durations, frequencies, and periods that exert the most significant influence on enhancing the overall healthy behavior of middle-aged and older adults remain subjects of ongoing exploration. More importantly, these moderator variables are not only essential for the development and implementation of exercise intervention strategies aimed at promoting health behaviors in middle-aged and older adults but also represent pivotal facets within the domain of exercise studies targeting health behavior promotion. Therefore, in order to address the aforementioned uncertainties, this study centers its intervention on exercise and adopts the principles of evidence-based medicine to try to identify key moderator variables associated with exercise intervention, so as to establish a novel foundation for the use of exercise in promoting health behaviors among middle-aged and older adults, as well as enhancing the scientific rigor and rationality of such approaches.

## 2 Research methods

### 2.1 Literature search

**FIGURE 1:**
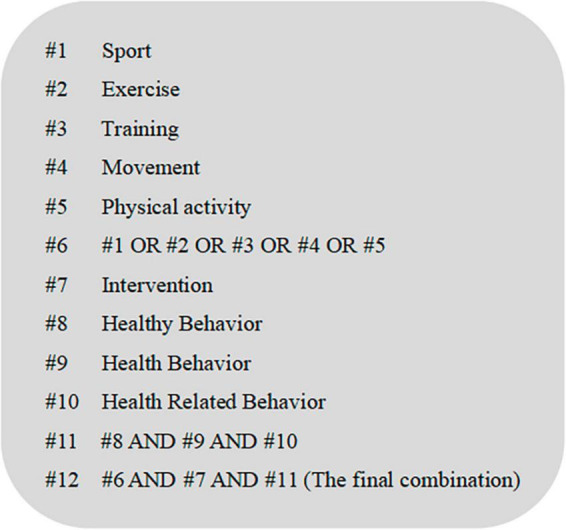
Search strategy of PubMed.

This systematic review followed the Preferred Reporting Items for Systematic Review and Meta-Analyses (PRISMA) to carry out literature search, identification, data extraction, and quality assessment, and combination of results. We conducted literature searches in both Chinese and English to maximize our search results. In the Chinese database, we utilized China National Knowledge Infrastructure (CNKI), Wanfang Database, and VIP Database for Chinese Technical Periodicals (VIP). In the English database, we employed Web of Science, PubMed, EBSCOhost, Embase, Elsevier, and the Cochrane Library. Our Chinese search terms included “tiyu (体 育),” “yundong (运 动),” “duanlian (锻 炼),” “shentihuodong (身 体 活 动),” “ganyu (干 预),” “jiankangxingwei (健 康 行 为),” and “jiankangxiangguanxingwei (健 康 相 关 行 为).” For our English search, we used terms including “sport,” “exercise,” “training,” “movement,” “physical activity,” “intervention,” “health behavior,” “healthy behavior,” and “health related behavior.” We employed “randomized controlled trial” as a qualifier or filter. In order to coincide with HFA/2000, which serves as a foundation for our systematic review, we imposed a limit to the articles published from 2000 and searched literature published from January 1^st^, 2000, to March 31^st^, 2023. Boolean operators were used. Figure 1 illustrates our search strategy in more detail as an example.

### 2.2 Literature inclusion and exclusion criteria

The inclusion criteria for quantitative systematic evaluation were developed based on the PICOS framework of evidence-based medicine, which is divided into five categories: Participants, Interventions, Comparisons, Outcomes, and Study Style (Yang et al., 2018). Based on the above 5 categories, we formulated the following literature inclusion and exclusion criteria.

Literature inclusion criteria: (1) Participants should be middle-aged and/or older adults with a minimum age or average age of 45 years old and above; (2) Exercise intervention should contain detailed information such as exercise type, exercise frequency, and duration of one exercise session, etc.; (3) Comparisons should involve routine interventions or blank controls; (4) Outcomes should measure the overall level of health behavior using scales, with all results quantitatively described; (5) The study design should be a Randomized Controlled Trial (RCT).

Literature exclusion criteria: (1) Excluded literature types encompass conference papers, dissertations, or non-experimental journal papers; (2) Literature languages not written in either Chinese or English; (3) Studies involving exercise teaching rather than exercise practices; (4) Literature with incomplete outcome descriptions; (5) Studies not adopting the RCT study design.

### 2.3 Literature identification, data extraction, and quality assessment

The searched bibliography was imported into NoteExpress, a reference management software, and the processes of literature screening, data extraction, and by using the Physiotherapy Evidence Database (PEDro) scale, the quality assessment were carried out back-to-back by 2 professional evaluators following the predefined inclusion and exclusion criteria.

#### 2.3.1 Literature identification

**FIGURE 2:**
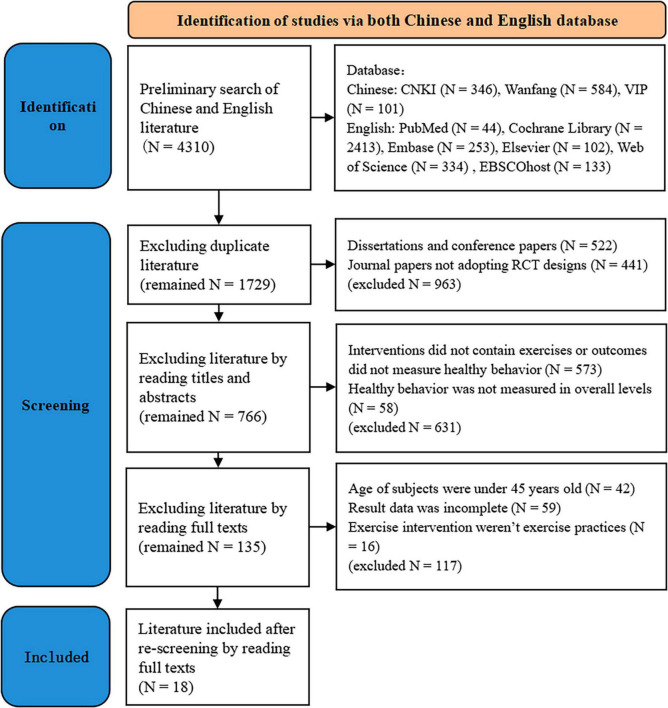
Flow chart of literature identification.

Literature meeting the inclusion criteria was identified from the searched bibliography. In case of disagreement between the evaluators, discussions or negotiations were facilitated with the assistance of a third evaluator. The literature identification process is carriout out according to the PRISMA 2020 flow diagram (Figure 2).

#### 2.3.2 Data extraction

Data extraction encompassed several key elements, including author names, publication dates, details of interventions (i.e., intervention means, intervention mode, exercise type, duration of one exercise session, exercise period, and exercise frequency), baseline information of subjects (i.e. subject type, sample size, gender, age), and outcome indexes, among others. As for those with incomplete information descriptions, attempts were made to contact the literature authors through e-mails to request supplementary details. If the responses were not received within 1 week, the respective literature were excluded.

#### 2.3.3 Quality assessment

The assessment was conducted using the PEDro scale, which has a total of 11 items. Each item meeting the specified criteria was assigned 1 point, with a score of 0 given for items not meeting the criteria. Notably, item 1 was excluded from the total score calculation. Quality assessment of the included literature was performed by 2 evaluators according to the PEDro scale. However, due to the inherent challenges in achieving blinding for all subjects and therapists who administered therapies in exercise intervention studies (Jiang et al., 2017), the blind assessment of this study was limited to blinding among assessors responsible for measuring at least one key outcome. A total score exceeding 4 indicated a relatively high level of literature quality.

### 2.4 Statistical analysis

In this study, the meta-analysis was conducted using the intervention review method in Review Manager 5.4.1 software (RevMan). Since each RCT included in the literature used a unique scale to evaluate the overall level of health behavior, the data analysis did not involve the selection of outcome values.

Firstly, the outcome values were subjected to standardization. Despite all data types being continuous, the evaluation scales utilized in each RCT varied. Therefore, to minimize errors, raw data underwent standardization before the meta-analysis. The standardization formula employed was as follows: standardized data = raw data/total score of the scale × 100.

Secondly, the inter-study heterogeneity was tested using the *I*^2^ statistics (with a significance level of 0.05). The criteria for judgment were as follows: *I*^2^ ≤ 25% indicated low-degree heterogeneity, 25% < *I*^2^ < 50% for moderate heterogeneity, and *I*^2^ ≥ 50% indicated high heterogeneity (Jiang et al., 2017; Yang et al., 2018). The standardized mean difference (SMD) known for its high consistency, served as the effect size indicator to optimize the analysis results (Wen and Li, 2007; Yang et al., 2018; Higgins et al., 2019). The criteria for judgment were as follows: SMD < 0.2 denoted a small effect size, 0.2 ≥ SMD < 0.5 indicated a moderate effect size, 0.5 ≤ SMD < 0.8 represented a medium effect size, and SMD ≥ 0.8 reflected a large effect size (Cohen, 1988). The random effect models was used for the combinations. The confidence interval (CI) used was set at 95%.

Thirdly, subgroup stratification was performed to analyze the sources of heterogeneity. Considering the basic elements included in the exercise intervention, the intervention means, intervention mode, exercise type, duration of one exercise session, exercise period, and exercise frequency were taken as the moderator variables to set subgroups. *I*^2^ statistics was the indicator to describe the inter-study heterogeneity and the random effect models was used to anticipate the heterogeneity in interventions and sample characteristics.

## 3 Research results

### 3.1 Results of the literature search and screening

In this study, a preliminary search yielded 4,310 literature (1,031 in Chinese and 3,279 in English). After the first exclusion of duplicate literature, 1,729 literature remained (857 in Chinese and 872 in English) were remained. By reading the titles and abstracts, 766 literature were retained after excluding dissertations, conference papers, and non-RCT literature. After examining the full texts, 135 literature were remained by the third exclusion based on inclusion criteria for interventions that did not contain exercise or for outcomes that were not overall levels of healthy behavior. Following a thorough re-screening process, 42 literature without middle-aged and older subjects, 59 literature without complete outcome results, and 16 literature without exercise practices were subsequently excluded. A total of 18 literature were included for analysis.

### 3.2 Basic characteristics of the included literature

A total of 18 RCT literature were included in this study. The key characteristics of the included literature were as follows:

(1) Intervention methods: In 15 literature, the interventions employed mixed approaches, combining exercise with other methods, while 3 literature solely utilized exercise as the intervention; Intervention modes: 5 literature involved group practices, 11 literature featured individual practices, and 2 literature employed a combination of group and individual practices; (2) Study participants: Among the articles, 15 focused on middle-aged and older patients, while 3 involved healthy (non-patient) middle-aged and older adults; In 2 literature, only female subjects were included, and 15 literature encompassed both male and female subjects (1 literature did not report gender); The total number of participants across all 18 literature was 1782, including 780 males and 912 females (1 literature did not report the number of men and women); Sample sizes ranged from 40 to 210 participants, with mean age spanning from 45 to 82 years. (3) Exercise intervention details: The exercise interventions encompassed various types, including aerobic exercise (i.e. walking, jogging, Tai Chi, wuqinxi), resistance exercise (involving self-weight and elastic bands) and stretching exercise (such as yoga, straight-leg raising, etc.). Intervention durations varied, ranging from 4 weeks to 1 year, with a most commonly adopted duration of 12 weeks. Single exercise sessions typically lasted 20 min or more, primarily falling within from the 30 to 60-min range. Exercise frequencies ranged from 2 to 7 times (days) per week, with most interventions involving 3 times (days) or less per week. (4) Comparisons: The compared interventions consisted of routine interventions or blank control groups. (5) Outcome measures: Health Promoting Lifestyle Profile (HPLP), Self-rated Abilities for Health Practices Scale (SRAHP), Health Behavior Questionnaire (HBQ), Health Behavior Scale (HBS) or Self-care of Heart Failure Index (SCHFI) were used to evaluate the overall level of health behaviors. Detailed information can be found in Table 1.

**TABLE 1:** Basic information of included literature.

References	Intervention means	Intervention mode	Subject type	Sample size (N)	Gender		Age (M ± SD)	Exercise type	Exercise time, frequency, period	Measurement
					**Male**	**Female**				
Dashtidehkordi et al., 2019	S (Exercise program by stationary bicycles)	I	Hemodialysis patients	EG = 27 CG = 25	6	46	EG: 51.22 CG: 55.64	Mini-bike	30 min cycling + 5 min rest, 2 rounds/time, 3 times/wk, 8 weeks	HPLP
Kim et al., 2021	C (LSIs based on King’s theory)	G	Healthy middle-aged women	EG = 26 CG = 30	0	56	EG: 48.19 ± 6.08 CG: 50.50 ± 6.35	Aerobic and stretching exercises	Walking 30 min/d (> 10,000 steps), Stretching > 2 groups/d, 7 d/wk, 8 weeks	HPLP
Mouodi et al., 2019	C (Lifestyle Interventions)	G	Healthy middle-aged adults	EG = 106 CG = 97	103	100	EG: 49.80 ± 5.20 CG: 49.50 ± 5.70	Aerobic activities	60 min/time, 2 times/wk, 16 weeks	HPLP
Saraboon et al., 2015	C (MUFIPs)	G	Older adults with Knee osteoarthritis	EG = 40 CG = 40	6	74	EG: 67.30 ± 6.30 CG: 67.5 ± 7.32	Aerobic and resistance exercises	Aerobic exercise: 1^st^ – 2^nd^ weeks (30 min/time), 3^rd^ – 4^th^ weeks (40 min/time), 5^th^ – 8^th^ weeks (60 min/time); 7 times/wk, 8 weeksResistance exercise: 10 times/round, 6 rounds/d, 7 d/wk, 8 weeks	HBQ
Song et al., 2007	S (Sun-style Tai Chi exercise program)	G/I	Older women with osteoarthritis	EG = 22 CG = 21	0	43	EG: 64.80 ± 6.00 CG: 62.50 ± 5.60	12 forms of Tai Chi	3–5 repetitions/time, 60 min/time; 1^st^ – 2^nd^ weeks (3 times/wk), 3^rd^ – 12^th^ weeks (3 – 4 times/wk); 12 weeks	HBS
Liu et al., 2022	C (Metformin combined with intensive-exercise diet therapy)	I	Renal cancer patients with diabetes mellitus	EG = 60 CG = 60	72	48	EG: 61.50 ± 11.25 CG: 59.28 ± 8.46	Moderate-intensity aerobic exercise	30–45 min/time, 6 times/wk, 1 weeks	HPLP-II
Zhang et al., 2018	C (Nurse-led transitional care programme)	G	Patients with coronary artery disease	EG = 100 CG = 99	107	92	EG: 66.60 ± 10.50 CG: 65.30 ± 8.13	Regular exercise recommended by pender	1–2 times/month, 7 months	SHARP
Gao et al., 2017	C (ADOPT)	G	Housebound elderly people	EG = 105 CG = 105	68	142	EG: 75.29 ± 7.52 CG: 77.28 ± 8.24	Traditional hua tuo medical wuqinxi	45 min/time; 1^st^ – 4^th^ wk (3 times/wk), 5^th^ – 26^th^weeks (5 times/wk); 26 weeks	HPLP
Gao et al., 2023	C (CRCI based on digital hospital)	I	Patients after PCI	EG = 45 CG = 45	57	33	EG: 67.36 ± 11.76 CG: 65.78 ± 11.48	Stretching and aerobic exercises	Within 3 days after PCI: 1 times/d; Discharge standard: > 30 min, > 4 times/wk; 1 month	SHARP
Guo et al., 2020	C (McMaster)	I	Patients with colorectal cancer	EG = 49 CG = 49	51	47	EG: 54.10 ± 6.20 CG: 54.20 ± 6.20	Interval aerobic exercise	15 min exercise + 5–10 min interval, 3 round/time; 60 min/time, 7 times/wk, 12 weeks	SRAHP
Li, 2015	C (Individualized health education)	I	Patients with hypertension	EG = 45 CG = 45	unreported		> 60 yrs	Aerobic exercise	30–40 min/time, 3–5 times/wk, 32 weeks	HPLP
Li et al., 2022	C (The whole course of rehabilitation management during the perioperative period based on Omaha theory)	I	TAVR patients	EG = 37 CG = 37	38	36	EG: 62.59 ± 5.03 CG: 63.21 ± 4.76	Aerobic exercise	20–30 min/time, 24 weeks	HPLP
Pan et al., 2022	C (Integrated interventions)	I	Patients with possible sarcopenia	EG = 34 CG = 30	41	23	EG: 71.59 ± 4.18 CG: 72.23 ± 5.28	Resistance exercise	45 min, 3 times/wk; 12 weeks	SRAHP
Wang Y. P et al., 2021	C (Diversified health education combined with personalized aerobic exercise)	unreported	Hypertension patients	EG = 40 CG = 40	51	29	EG: 46.55 ± 4.72 CG: 46.50 ± 4.68	Brisk walking	45–60 min/time, 3 times/wk, 12 weeks	HPLP
Wang Q. Z et al., 2019	C (Health education based on TTM)	I	Patients with chronic heart failure	EG = 43 CG = 43	50	36	EG: 68.99 ± 4.11 CG: 69.35 ± 3.74	Aerobic exercise	1^st^ – 2^nd^ weeks, 20 min/time, 1 times/d; 3^rd^ – 4^th^ weeks, 30 min/time, 2 times/d; 3–4 d/wk (alternate days), 4 weeks	SCHFI
Wang Y. N et al., 2022	S (Baduanjin exercise mode based on self-regulation theory)	G/I	Healthy older adults	EG = 32 CG = 32	18	46	EG: 81.69 ± 6.71 CG: 81.19 ± 5.38	Baduanjin	20–30 min/time, 4 times/wk, 6 cycles, 12 weeks	HPLP-C
Wang X. R et al., 2022	C (Hospital-community-family integrated model)	I	Patients with chronic heart failure	EG = 41 CG = 40	58	23	EG: 54.41 ± 13.93 CG: 56.10 ± 13.01	Aerobic exercise	Cycling: 1–2 times/wk, 35–60 min/time, 6 months; Walking: 2 times/wk, 35–60 min/time, 6 months	HPLP-II
Wang et al., 2023)	C (Healthy behavior intervention based on HAPA)	I	Patients with ACOS	EG = 46 CG = 46	54	38	EG: 61.57 ± 3.64 CG: 60.85 ± 3.25	Aerobic and resistance exercises	1 time/d, 6 months	HPLP

(1) EG: Experimental group; CG: Control group; M: Mean; SD: Standard deviation. (2) G: Group practice; I: Individual practice; G/I: Group/individual practice. (3) C: Combined regimen including exercise; S: Singular approach (only exercise). (4) HPLP: Health Promoting Lifestyle Profile; SRAHP: Self-rated Abilities for Health Practices Scale; HBQ: Health Behavior Questionnaire; HBS: Health Behavior Scale; SCHFI: Self-care of Heart Failure Index. (5) LSIs: Lifestyle interventions; MUFIPs: Multifactorial intervention programs; ADOPT: Attitude-definition-open-plan-try it out intervention program; McMaster: McMaster family therapy; CRCI: Cardiac rehabilitation comprehensive intervention; TTM: Transtheoretical model of change. (6) ACOS: Asthma COPD (chronic obstructive pulmonary diseases) overlap syndrome; PCI: Percutaneous coronary intervention; TAVR: Transcatheter aorticvalve replacement.

### 3.3 Results of the quality assessment of the included literature

By assessing the quality results of the 18 literature with PEDro (Table 2), the average score of the included literature was 5.89. Individual score of each literature ranged from 4 to 8. These scores collectively indicate that all the included literature exhibited a high level of quality.

**TABLE 2:** PEDro scores of the included literature.

References	Eligibility criteria	Random allocation	Concealed allocation	Similar baseline	All assessors were blinded	Measures of at least one key outcome were obtained from more than 85% of the subjects	Intention to treat	Between-group statistical comparison report	Both point measures and measures of variability for at least one key outcome	Total score
Dashtidehkordi et al., 2019	1	1	1	1	0	1	0	1	1	6
Kim et al., 2021	1	1	0	1	0	1	0	1	1	5
Mouodi et al., 2019	1	1	1	1	0	1	1	1	1	7
Saraboon et al., 2015	1	1	1	1	0	1	1	1	1	7
Song et al., 2007	1	1	0	1	0	1	0	1	1	5
Liu et al., 2022	1	1	0	1	0	1	1	1	1	6
Zhang et al., 2018	1	1	1	1	1	1	1	1	1	8
Gao et al., 2017	1	1	0	1	0	1	0	1	1	5
Gao et al., 2023	1	1	0	1	0	1	1	1	1	6
Guo et al., 2020	1	1	0	1	0	1	1	1	1	6
Li, 2015	1	1	0	1	0	1	1	1	1	6
Li et al., 2022	1	1	0	1	0	1	1	1	1	6
Pan et al., 2022	1	1	0	1	0	1	1	1	1	6
Wang Y. P et al., 2021	1	1	0	1	0	1	1	1	1	6
Wang Q. Z et al., 2019	1	1	0	1	0	1	1	1	1	6
Wang Y. N et al., 2022	1	0	0	1	0	1	0	1	1	4
Wang X. R et al., 2022	1	1	0	1	0	1	1	1	1	6
Wang et al., 2023	1	0	0	1	0	1	1	1	1	5

### 3.4 Results of risk and sensitivity analysis on publication bias of the included literature

**FIGURE 3:**
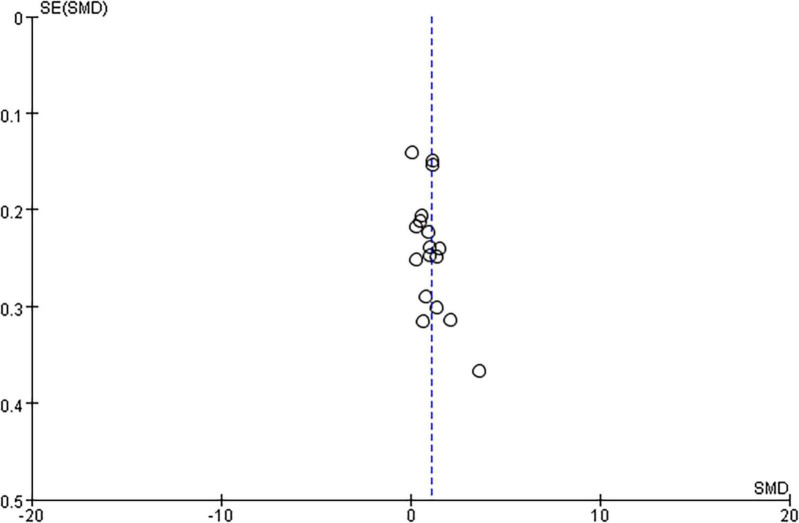
Funnel plots of publication bias.

The funnel plot is a commonly employed method to identify publication bias. It is constructed as a scatter plot with effect size on the X-axis and sample size (or the reciprocal of the standard error of the effect size) on the Y-axis. Typically, when testing for publication bias, the minimum number of included literature is should not be less than 9 (Jiang et al., 2017). In this study, we included a title of 18 literature, allowing for a robust test for publication bias. As depicted in Figure 3, there was a slight deviation in the distance of one literature from the others, indicating some heterogeneity. However, the scatter distribution of the remaining literature was symmetrical on both sides of the axis, indicating the absence of significant publication bias.

Sensitivity analysis is one of the valuable approaches for addressing heterogeneity by estimating the robustness of the results by manipulating the factors that may affect the results. In this study, the results exhibited minimal alterations when subjected to re-analysis involving changes in inclusion order and effect model. This consistency underscores the low sensitivity of the findings, affirming the stability and credibility of the meta-analysis results in this study.

### 3.5 Results of the meta-analysis

#### 3.5.1 The overall effect test of exercise intervention

**FIGURE 4:**
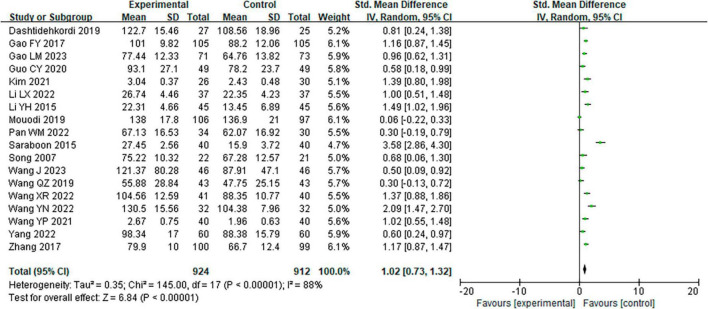
Forest plot of the overall effect of exercise intervention on healthy behavior in middle-aged and older adults.

Through the overall effect test (Figure 4), it was found that the merged effect size of exercise intervention on health behavior was statistically significant [SMD = 1.02 > 0.8, 95% CI (0.73, 1.32), two-tailed test Z = 6.75, *P* < 0.00001], indicating that the exercise intervention had a significant effect on health behavior among middle-aged and older adults.

According to the results from the overall heterogeneity test, *I*^2^= 88.0% > 50%, *P* < 0.00001, prompting the utilization of a random effects model for effect size amalgamation. In the meta-analysis encompassing the 18 included literature, the heterogeneity among multiple groups of data was high. This highlights the potential influence of moderator variables on the overall effect size, and subgroup analysis was needed. To mitigate the influence of the subject differences, one subgroup analysis was performed according to whether the subjects were patients or not. The results showed that *I*^2^ = 41.7% < 50%, *P* = 0.11 > 0.05, rulling out the possibility of heterogeneity in the overall effect of exercise intervention being attributed to differences in subject population types.

#### 3.5.2 Subgroup analysis of moderator variables

Subgroup analysis is an significant approach for analyze the causes of heterogeneity. In the subgroup analysis, the fewer subgroups, the more robust (Yang et al., 2018). The number of each subgroup was less 4 (Table 3).

**TABLE 3:** Results of subgroup analysis of moderator variables on health behavior in middle-aged and older adults.

Moderator Variable	Subgroup	Number of literature	Sample size	Heterogeneity test			Two-tailed test		Weight	SMD, 95%CI
				**Chi^2^**	***I* ^2^**	**P**	**Z**	**P**		
Intervention mode	Total	18	1782	0.13	0.0%	0.72	6.79	< 0.00001	100.0%	1.05 [0.74, 1.35]
	Combined regimen including exercise	15	1623				6.09	< 0.00001	84.60%	1.02 [0.69, 1.35]
	Singular approach (only exercise)	3	159				2.69	0.007	15.40%	1.19 [0.32, 2.06]
Intervention means	Total	17	1690	1.97	0.0%	0.37	6.68	< 0.00001	100.0%	1.08 [0.76, 1.40]
	Group/individual	2	107				1.96	0.05	10.80%	1.38 [0.00, 2.77]
	Group	5	748				3.39	0.0007	29.80%	1.42 [0.60, 2.24]
	Individual	10	835				6.35	< 0.00001	59.30%	0.87 [0.60, 1.14]
Exercise type	Total	18	1782	1.52	0.0%	0.68	6.79	< 0.00001	100.0%	1.05 [0.74, 1.35]
	Aerobic and stretching	2	146				5.37	< 0.00001	10.90%	1.13 [0.72, 1.54]
	Aerobic and resistance	2	172				1.32	0.19	10.50%	2.03 [-0.99, 5.05]
	Resistance	2	128				1.32	0.19	10.60%	1.18 [-0.57, 2.94]
	Aerobic	12	1336				5.98	< 0.00001	68.10%	0.88 [0.59, 1.16]
Exercise period	Total	18	1782	2.66	0.0%	0.45	6.79	< 0.00001	100.0%	1.05 [0.74, 1.35]
	4 weeks – 1 month	2	176				1.89	0.06	11.40%	0.63 [-0.02, 1.28]
	8–12 weeks	8	537				3.97	< 0.0001	42.20%	1.28 [0.65, 1.91]
	16 weeks – 6 months	4	450				2.32	0.02	22.80%	0.80 [0.12, 1.48]
	26 weeks –1 year	4	619				6.68	< 0.00001	23.60%	1.09 [0.77, 1.42]
Duration of one exercise session	Total	17	1589	6.95	71.7%	0.03	6.33	< 0.00001	100.0%	1.04 [0.73, 1.36]
	> 60 min	4	396				2.42	0.02	23.6%	0.48 [0.09, 0.87]
	30–60 min	10	969				5.81	< 0.00001	59.2%	1.20 [0.80, 1.61]
	20–30 min	3	224				2.34	0.02	17.3%	1.23 [0.20, 2.27]
Exercise frequency	Total	17	1708	1.46	0.0%	0.48	6.42	< 0.00001	100.0%	1.03 [0.71, 1.34]
	< 3 time (d)/wk	6	679				3.19	0.001	36.00%	0.78 [0.30, 1.26]
	3–5 time (d)/wk	6	583				4.88	< 0.00001	35.10%	1.10 [0.66, 1.54]
	6–7 time (d)/wk	5	446				3.00	0.003	28.90%	1.29 [0.45, 2.13]

(1) Intervention means

The intervention means encompassed physical exercise as either a singular approach or as a part of a combined regimen. This moderator variable included a sample size of 1782, *I*^2^= 0%, *P* > 0.05, with no heterogeneity. The singular approach (only exercise) had the largest effect size [SMD = 1.19 (0.32, 2.06), *P* = 0.007], followed by the combined regimen including exercise [SMD = 1.02 (0.69, 1.35), *P* < 0.00001].

(2) Intervention mode

The intervention mode refers to the mode of exercise practices, which were divided into the group practice and the individual practice in this study. This moderator variables included a sample size of 1,690 (one literature did not report it), *I*^2^= 0%, *P* > 0.05, with no heterogeneity. The group practice mode had the largest effect size [SMD = 1.42 (0.60, 2.24), *P* = 0.0007]; followed by the group/individual practice mode [SMD = 1.38 (0.00, 2.77), *P* = 0.05]; and the individual practice mode yielded the smallest effect size [SMD = 0.87 (0.60, 1.14), *P* < 0.00001].

(3) Exercise type

The exercise type refers to various forms of exercise practices which were divided into the aerobic exercise, the resistance exercise, and the stretching exercise. This moderator variable included a sample size of 1782, *I*^2^= 0%, *P* > 0.05, with no heterogeneity. The combination of aerobic and stretching exercises had the highest effect size [SMD = 1.13 (0.72, 1.54), *P* < 0.00001], followed by aerobic exercise [SMD = 0.88 (0.59, 1.16), *P* < 0.00001].

(4) Exercise period

The exercise period refers to the whole duration of exercise practice. This moderator variable included a sample size of 1,782, *I*^2^= 0%, P > 0.05, with no heterogeneity. The exercise period ranging from 8 weeks to 12 weeks had the highest effect size [SMD = 1.28 (0.65, 1.91), *P* < 0.0001], followed by the exercise period ranging from 26 weeks to 1 year [SMD = 1.09 (0.77, 1.42), *P* < 0.00001].

(5) Duration of one exercise session

The duration of one exercise session refers to the length of one exercise time. This moderator variable included a sample size of 1,589 (one article did not report it), *I*^2^= 71.7% > 50%, *P* < 0.05. The high heterogeneity indicated that the duration of one exercise session can influence the relationship between exercise intervention and health behavior, and it is the cause of the heterogeneity in the overall effect of exercise intervention. The duration of one exercise session from 20 to 30 min had the highest effect size [SMD = 1.23 (0.20, 2.27), *P* = 0.02]; followed by the duration of one exercise session from 30 to 60 min [SMD = 1.20 (0.80, 1.61), *P* < 0.00001]; The duration of one exercise session of over 60 min had the smallest effect size [SMD = 0.48 (0.09, 0.87), *P* = 0.02].

(6) Exercise frequency

The Exercise frequency refers to the number of days or times of exercise practice per week. This moderator variable included a sample size of 1,708 (one article did not report it), *I*^2^= 0%, *P* > 0.05, with no heterogeneity. The effect size could be larger with the increase of exercise frequency. The Exercise frequency from 6 to 7 times (days) per week had the highest effect size [SMD = 1.29 (0.45, 2.13), *P* = 0.003]; The exercise frequency of less than 3 times (days) per week had the smallest effect size [SMD = 0.78 (0.30, 1.26), *P* = 0.0015].

## 4 Discussion

Exercise intervention is a significant way to promote healthy behaviors, fostering health intentions and cultivating healthy habits (Qi and Mao, 2018). The study results showed that the merged effect size of exercise intervention was 1.02 (*P* < 0.05), indicating that exercise intervention significantly promoted the health behavior of middle-aged and elderly adults. According to the theory of information-motivation-behavior kill model (Fisher et al., 1996), behavioral changes hinge on motivation, and health behavior changes when interventions are consistently implemented and accumulated sufficiently. In this context, physical exercise plays a pivotal role in bolstering participants’ motivation to engage in the intervention process and achieve their defined health goals (Gao, 2015). The meta-analysis results not only supported the conclusion that exercise intervention promoted healthy behavior in middle-aged and elderly adults, but also found out the key moderator variable of exercise intervention affecting on the overall level of health behaviors. The study found that the overall heterogeneity was high (*I*^2^= 88%). According to the results of heterogeneity test, it was found that only the *I*^2^ of the duration of one exercise session was significantly higher than 50%, the *I*^2^ of other moderator variables were all 0% (*P* > 0.05). It showed that the duration of one exercise session was the reason for the heterogeneity, indicating that it is the most critical moderator variable.

In terms of the intervention means and mode, while 15 out of the 18 included literature employ the combined regimen including exercise, from the results of the effect size values, we can see that the singular approach (only exercise) has the largest effect size, followed by the combined regimen including exercise. Thus, this implies the singular approach (only exercise) has better potential effect on helping promote healthy behaviors at least to some extent. However, it is needed to be further explored considering the risk of bias because only 3 RCTs were included in the singular approach (only exercise) subgroup (Gong et al., 2020). The mode of group practice had the largest effect size, which may be related to the peer effect and organizational management during group practice. Peer effect refers to the degree to which the behaviors and relationships of peers influence their own behaviors. Prior studies showed that the formation of fitness behavior consciousness is positively correlated with peer relationship and the degree of organization of exercise activities (Wang F. B. H et al., 2019). During the group practice, the participants were organized to exercise in a group with the behavior model and support of peers, encouragement from their coaches or teachers, and supervision by the organizers, contributing to mobilizing the enthusiasm in exercise participation, improving the degree of exercise practice, and developing the formation of healthy behavior habits.

As for the exercise type, the results of meta-analysis favored that the combination of aerobic and resistance exercise had the highest effect size, following the subgroup of resistance exercise, while only 2 RCTs were included in each subgroup, which might implicate some biases (Gong et al., 2020). It is well known that both aerobic and resistance exercise programs are important to promote general health. The aerobic subgroup included 12 RCTs, and aerobic exercise, on the other hand, holds a distinct advantage in daily life due to its commonality, lower injury risk owing to its moderate intensity, and particular suitability for cardiorespiratory fitness. Among the 18 literature included in this study, the subjects in 14 literature were patients with disease, of which 65.1% were patients with cardiovascular or respiratory disease. Middle-aged and elderly patients mostly participate in exercise practice for the purpose of increasing rehabilitation and treatment effects, and do not want to suffer from secondary injuries, so the aerobic exercise is one of the most safe choice. Cardiorespiratory fitness is a core element of physical fitness. Prior evidences have shown that low-level cardiorespiratory fitness was closely associated with cardiovascular disease risk, all-cause mortality, and cancer mortality, and had stronger predictive power of mortality than those established risk factors, such as smoking, high blood pressure, and high cholesterol(Lin et al., 2012; Ross et al., 2016). Previous studies have found that aerobic exercise has a more pronounced effect on lowering blood pressure in middle-aged and elderly patients with hypertension than resistance exercise (Yang, 2018). In the meta-analysis of the impact of bone mineral density on middle-aged and elderly adults, it was also found that aerobic exercise produced the most significant improvement (Dong et al., 2016). Xu et al. (2020) also found that aerobic exercise was the best exercise type to improve health. Therefore, no matter aerobic or resistance exercise, they both help to promote health behavior but greater results may be achieved with the combined of them.

In the fields of the exercise period, duration of one exercise session, and exercise frequency, this study found that although the duration of one exercise session ranging from 20 to 30 min (3 literature included) had the largest effect size on promoting health behaviors, followed by the duration of one exercise session ranging from 30 to 60 min (10 literature included), the difference of the effect size gap between the two subgroups is very small (the difference is 0.03). Therefore, considering the bias in conclusions drawn from very few studies (Gong et al., 2020), it can be concluded that the duration of one exercise session ranging from 30 to 60 min is better than others. The exercise frequency of 6–7 times (days) a week and the exercise period from 8 to 12 weeks both had the largest effect sizes. Dong et al. (2016) has pointed out that the aerobic exercise with 30–60 min one time, more than 3 times per week can help improve the health quality of middle-aged and elderly adults. Fang et al. (2020) also found that the exercise frequency of hypertensive patients was usually 3–5 times a week with 30–60 min a time through the meta-analysis. Despite this study found that the effect size became progressively larger with the prolonged exercise frequency, which was in line with Cai et al. (2022), 6–7 times/week may not be a real deal for some middle-age and older adults. Consequently, considering that < 3 and 3–5 weekly frequencies were also significant (even with small effect sizes), starting with a lower weekly frequency can also be beneficial, but greater results may be achieved with increasing frequency.

Although the 18 RCTs included in this study have high quality and low sensitivity, we should acknowledge the existence of a high heterogeneity within interventions and sample populations. Thus, we need to consider in the light of the limitations from the existing literature. Several limitations merit consideration. First, the number of included RCTs and the sample size were small, with only 18 RCTs and a sample size of 1782. Despite the exhaustive search of databases and literature forms was conducted during the literature search, the possibility of missing literature was not ruled out. If the sample size and the number of RCTs are large enough, the results will be more applicable. Second, although the randomized control, grouping and data results were introduced in all the 18 RCTs, some descriptions in few RCTs were incomplete, such as the lack of complete introduction of moderator variables and the specific implementation of blinding. Therefore, in the subgroup analysis, the situation exist that only 2 RCTs in some subgroups, resulting in a certain bias. Third, in selecting health behavior indicators, our study exclusively focused on evaluating the overall level of health behavior, without exploring the various dimensions of health behavior.

The systematic review is mainly focused on the overall effect of exercise intervention on health behavior, and the sub-group analysis is only an additional result that provide some insights, which should be highlighted the need of caution in interpretating and generalizing the results to all middle-aged and older adults considering the heterogeneity among interventions and samples, and in turn, should be further explored. Additionally, 15 out of 18 included literature employ exercise in combination with other methods. The effect of different combinations should also be discussed in the future studies. Therefore, in the future studies, firstly, it is advisable to expand the scope of literature inclusion to enhance the robustness of future research in this area. This can be achieved by broadening the range of databases searched and incorporating literature in languages other than Chinese and English. These measures will contribute to an increased number of studies and larger sample size. Secondly, future studies should delve deeper into the effects of different intervention programs including different combinations means.

## 5 Conclusion

(1) Exercise intervention exhibit a substantial and highly positive effect size when it comes to promoting the health behavior among middle-aged and older adults. Notably, the duration of one exercise session emerges as the key moderator variable contributing to this heterogeneity. (2) The optimal exercise intervention should include the means of group practice, the types of aerobic and resistance exercise, with a duration of 30–60 min per session, starting with a lower weekly frequency and gradually increase to 6–7 times per week, and spanning 8 to 12 weeks.

## Data availability statement

The raw data supporting the conclusions of this article will be made available by the authors. Requests to access these datasets should be directed to GC, chengong@pku.edu.

## Author contributions

ML: Data curation, Formal analysis, Resources, Writing−original draft. D-HM: Data curation, Formal analysis, Resources, Writing−review and editing. Y-LZ: Funding acquisition, Project administration, Writing−review and editing. NK: Data curation, Formal analysis, Writing−review and editing. D-MW: Conceptualization, Funding acquisition, Project administration, Supervision, Writing−review and editing. GC: Conceptualization, Funding acquisition, Project administration, Supervision, Writing−review and editing.
